# Mutation load decreases with haplotype age in wild Soay sheep

**DOI:** 10.1002/evl3.229

**Published:** 2021-05-17

**Authors:** Martin A. Stoffel, Susan E. Johnston, Jill G. Pilkington, Josephine M. Pemberton

**Affiliations:** ^1^ School of Biological Sciences, Institute of Evolutionary Biology University of Edinburgh Edinburgh EH9 3FL United Kingdom

**Keywords:** Deleterious mutation, fitness, inbreeding depression, ROH, runs of homozygosity, survival

## Abstract

Runs of homozygosity (ROH) are pervasive in diploid genomes and expose the effects of deleterious recessive mutations, but how exactly these regions contribute to variation in fitness remains unclear. Here, we combined empirical analyses and simulations to explore the deleterious effects of ROH with varying genetic map lengths in wild Soay sheep. Using a long‐term dataset of 4879 individuals genotyped at 417K SNPs, we found that inbreeding depression increases with ROH length. A 1% genomic increase in long ROH (>12.5 cM) reduced the odds of first‐year survival by 12.4% compared to only 7.7% for medium ROH (1.56–12.5 cM), whereas short ROH (<1.56 cM) had no effect on survival. We show by forward genetic simulations that this is predicted: compared to shorter ROH, long ROH will have higher densities of deleterious alleles, with larger average effects on fitness and lower population frequencies. Taken together, our results are consistent with the idea that the mutation load decreases in older haplotypes underlying shorter ROH, where purifying selection has had more time to purge deleterious mutations. Finally, our study demonstrates that strong inbreeding depression can persist despite ongoing purging in a historically small population.

**Impact Statement**

The harmful consequences of inbreeding have fascinated scientists since Darwin, but only recently have genomic tools allowed us to study the underlying genetic causes. We know now that the reduction in fitness in inbred individuals, termed inbreeding depression, is due to increased homozygosity across the genome. This causes more deleterious recessive mutations to express their effects, thereby affecting health, fertility, and survival. Inbreeding depression can be particularly problematic for wild species with small population sizes, where inbreeding is common and can accelerate extinction. Yet, the complex relationship between inbreeding depression, deleterious mutations and their removal by natural selection is not well understood in wild populations.

Here we studied the genetics of inbreeding depression in wild Soay sheep, a primitive breed that has lived largely unmanaged on the Scottish St. Kilda archipelago for thousands of years. All individuals, but in particular inbred individuals, carried many ROH, which are continuous stretches of homozygous genotypes. We found that long ROH reduced the probability of an individual surviving its first winter disproportionately more than short ROH, because long ROH are enriched for deleterious mutations. Using detailed genetic simulations of our population where we tracked every mutation in each individual, we found that this pattern emerges because natural selection constantly removes strongly deleterious mutations from the population, leaving older and shorter ROH with fewer mutations. Overall, our study provides a glimpse into the complex interplay between individual fitness, deleterious mutations and selection against these mutations in a wild mammal population.

## Introduction

The structure of deleterious genetic variation in natural populations shapes a range of processes in evolutionary biology, such as the strength of inbreeding depression and the efficiency of genetic purging (Charlesworth and Willis, 2009; Hedrick and Garcia‐Dorado, 2016). The role of deleterious mutations is also increasingly discussed in applied conservation, in particular when considering genetic rescue of small populations (Kyriazis et al., 2021; Ralls et al., 2020). To date, studies in wild populations have mostly focused on average, genome‐wide fitness effects of deleterious recessive alleles through measuring genome‐wide inbreeding coefficients (Hoffman et al., 2014; Bérénos et al., 2016; Chen et al., 2016; Huisman et al., 2016; Harrisson et al., 2019; Niskanen et al., 2020), or genome‐sequence based predictions of deleterious mutations (Xue et al., 2015; Robinson et al., 2018; Grossen et al., 2020). Therefore, we still know very little about how deleterious mutations in different parts of the genome contribute to inbreeding depression, as these analyses usually require large samples of individuals with known fitness and dense genomic data—both of which are scarce in wild nonmodel organisms.

In populations for which mapped genetic markers are available, runs of homozygosity (ROH) open up new possibilities for studying the effects of (partially) recessive deleterious variation. These long stretches of homozygous genotypes are ubiquitous in diploid genomes and commonly arise when individuals inherit homologous haplotypes which are identical by descent (IBD), originating from a single copy of the region in a common ancestor. Offspring of related parents have more ROH, which in turn increases the probability that partially recessive deleterious alleles are expressed, thereby causing inbreeding depression (Charlesworth and Willis, 2009). The lengths and numbers of ROH can vary widely between individuals, and have been shown to contribute to the genetic architecture of complex traits and diseases in humans (Ceballos et al., 2018; Clark et al., 2019) and to production traits in livestock (Pryce et al., 2014; Ferenčaković et al., 2017). In wild populations, ROH are increasingly used to precisely measure individual inbreeding coefficients (Kardos, Luikart, and Allendorf, 2015; Kardos et al., 2018) and the effects of inbreeding on fitness (Stoffel et al., 2021b; Bérénos et al., 2016). Moreover, genome‐wide association studies (GWAS) are starting to uncover associations between ROH at specific locations in the genome and complex traits or fitness, thereby providing information about the distribution of effect sizes at loci causing inbreeding depression (Stoffel et al., 2021b; Pryce et al., 2014).

The length of an ROH allows one to estimate the time to a most recent common ancestor (MRCA) of the underlying IBD haplotypes (Thompson, 2013). In any given generation, DNA is inherited in physically large chunks with genetic map lengths of around 100 centimorgan (cM), and recombination breaks up these segments in successive generations. For example, an initial segment is broken up into smaller IBD segments with an expected length of 2 cM after 25 generations, or 50 meioses (Thompson, 2013). The expected genetic map length (*L*) of an ROH can be estimated as *L* = 100/(2 × *g*) cM, where *g* is the number of generations to the MRCA (Thompson, 2013), though the distribution is exponential with high variance due to stochastic effects of recombination and Mendelian segregation (Thompson, 2013; Kardos et al., 2016). Long ROH originating from close inbreeding are expected to have a recent ancestor, whereas short ROH have an ancestor further back in the pedigree. In addition, when the effective size (*N*_e_) of a population has been small at a given point in the recent history, ROH with an MRCA at that point will be more abundant in the current population. The relative frequencies of ROH of different lengths are therefore informative about recent fluctuations in population sizes (Browning and Browning, 2015; Kardos, Qvarnström, and Ellegren, 2017; Ceballos et al., 2018).

Considering jointly the fitness effects and sizes of ROH allows us to investigate how ROH lengths (and therefore haplotype ages) are associated with inbreeding depression and mutation load. Given that ROH lengths provide an expectation for the number of generations for which the underlying haplotypes have been exposed to selection, we hypothesize the following: Short ROH originating further back in the pedigree should be depleted of deleterious recessive variation, as purifying selection has had many generations to remove these mutations. In contrast, long ROH emerging from younger haplotypes should on average carry larger numbers of strongly deleterious recessive mutations at lower frequencies, and therefore be associated with stronger effects on fitness. In humans, ROH in general and especially long ROH are enriched for mutations which are predicted to be deleterious (Pemberton and Szpiech, 2018; Szpiech et al., 2019, 2013), but to our knowledge, these predictions have not been tested using actual fitness data in a wild population. Quantifying the fitness effects of different ROH length classes could help to understand the genetic basis of inbreeding depression and provide a novel way to assess the efficiency of selection against deleterious mutations in wild populations.

Here, we combined long‐term life‐history data for 4879 wild Soay sheep with 417K SNP genotypes and linkage map information to test whether inbreeding coefficients (*F*_ROH_) calculated from ROH with long, medium, and short genetic map lengths differ in their contribution to inbreeding depression in first‐year survival. We then used forward genetic simulations based on the Soay sheep demographic history to quantify the expected differences in the mutation load among ROH length classes and to explore the underlying causes. We discuss how our results fit into current knowledge about inbreeding depression and purging in small populations and methodological implications for studying the genetic basis of inbreeding depression.

## Materials and Methods

### STUDY POPULATION

Soay sheep are descendants of primitive European domestic sheep and have lived unmanaged on the St. Kilda archipelago, Scotland, for thousands of years (Clutton‐Brock and Pemberton, 2004). A part of the population in the Village Bay area on the island of Hirta (57°49’N, 8°34’W) has been the focus of a long‐term individual‐based study since 1985 (Clutton‐Brock and Pemberton, 2004). More than 95% of individuals in the study area are ear‐tagged within a week after birth during the lambing season from March to May, and DNA samples are obtained from either blood samples or ear punches. Routine mortality checks, in particular during peak mortality at the end of winter, usually find around 80% of deceased animals (Bérénos et al., 2016). Here, we focused on the fitness trait “first‐year survival”, where every individual was given a 1 if it survived from birth (March to May) to the April 30 of the next year, and a 0 if it did not, with measures available for 4879 individuals born from 1979 to 2018. To impute genotypes, we assembled a pedigree based on 438 unlinked SNP markers from the Ovine SNP50 BeadChip using the R package Sequoia (Huisman, 2017). In the few cases where no SNP genotypes were available, we used either observations from the field or microsatellite markers (Morrissey et al., 2012). All animal works were carried out according to UK Home Office procedures and were licensed under the UK Animals (Scientific Procedures) Act of 1986 (Project License no. PPL70/8818).

### GENOTYPING

We genotyped a total of 7700 Soay sheep on the Illumina Ovine SNP50 BeadChip. We used the check⋅marker function in GenABEL version 1.8‐0 (Aulchenko et al., 2007) for quality control, filtering for SNPs with minor allele frequency >0.001, SNP locus genotyping success >0.99, individual genotyping success >0.95, and identity by state with another individual <0.9. We also genotyped 189 sheep on the Ovine Infinium HD SNP BeadChip, resulting in 430,702 polymorphic SNPs for 188 individuals, after removing monomorphic SNPs, and filtering for SNPs with SNP locus genotyping success >0.99 and individual sheep with genotyping success >0.95. These sheep were specifically selected to maximize the genetic diversity represented in the full population (for full details, see Johnston et al., 2016). All SNP positions were based on the Oar_version 3.1 sheep reference genome, a chromosomal‐level assembly with 26 autosomes, one X chromosome, and a scaffold N50 of 100 Mb (GenBank assembly ID GCA_000298735.1; Jiang et al., 2014).

### GENOTYPE IMPUTATION

The detailed genotype imputation methods are presented elsewhere (Stoffel et al., 2021b). Briefly, we first merged the datasets from the 50k SNP chip and from the HD SNP chip with—bmerge in PLINK version 1.90b6.12 (Purcell et al., 2007), resulting in a dataset with 436,117 SNPs including 33,068 SNPs genotyped on both SNP chips. We then discarded SNPs on the X chromosome and focused on the 419,281 SNPs located on autosomes. To impute SNPs with genotypes missing in individuals genotyped at the lower SNP density, we used AlphaImpute version 1.98 (Hickey et al., 2012), which uses both genomic and pedigree information for phasing and subsequent imputation of missing genotypes. After imputation, we filtered SNPs with call rates below 95%. Overall, this resulted in a dataset with 7691 individuals, 417,373 SNPs, and a mean genotyping rate per individual of 99.5% (range 94.8%–100%). We evaluated the accuracy of genotype imputation using 10‐fold leave‐one‐out cross‐validation. In each iteration, we randomly chose one individual genotyped on the high‐density (HD) SNP chip, masked genotypes unique to the HD chip, and imputed the masked genotypes. This allowed us to compare the imputed genotypes to the true genotypes and to evaluate the accuracy of the imputation. Overall, 99.3% of genotypes were imputed correctly. Moreover, the distribution of inbreeding coefficients *F*_ROH_ was very similar for individuals genotyped on the HD chip and individuals with imputed SNPs, indicating little difference in inferred ROH between the two groups and hence no obvious bias in ROH calling based on imputed genotypes (Stoffel et al., 2021b).

### INFERRING LINKAGE MAP POSITIONS

We used a dense, sex‐averaged Soay sheep linkage map with 36,972 autosomal markers (Johnston et al., 2016) to infer the genetic map positions in cM for each SNP in the imputed dataset. As the imputed SNP dataset used here had a higher SNP density than the linkage map SNP dataset, we interpolated the genetic positions of SNPs that were not present in the linkage map dataset by assuming a constant recombination rate in genomic regions between linkage mapped SNPs (Kardos et al., 2017, 2018). If two flanking SNPs had the same coordinates on the genetic map, all imputed SNPs in between were assigned the same genetic map position. If two flanking SNPs had different genetic map positions, the SNPs in between were assigned increasing genetic map positions depending on the physical distance to each of the two SNPs. For example, if the two flanking SNPs had cM positions 3 and 4, an imputed SNP half way between these SNPs on the physical map got assigned a cM position 3.5. Imputed SNPs occurring before the first linkage mapped SNP on a chromosome were assigned a genetic map position of 0 cM, and SNPs occurring after the last linkage mapped SNP on a chromosome were assigned the same genetic position as the last linkage mapped SNP.

### ROH CALLING AND INDIVIDUAL INBREEDING COEFFICIENTS *F*_ROH_

We focused on ROH quantified by their genetic map lengths rather than physical map lengths as this accounts for the effects of recombination rate variation on detected ROH lengths (Kardos et al., 2017) and allows us to infer an expected time of coalescence for each ROH more precisely, assuming ROH are true IBD segments (Thompson, 2013). To call ROH based on their genetic map lengths in cM, we used PLINK (Purcell et al., 2007), replacing physical with genetic map positions in the input *.map* file. To keep the parameter arguments on a comparable scale to running PLINK with base‐pair positions, we multiplied cM positions by 1 × 10^6^. We then called ROH with a minimum length of 0.39 cM containing at least 25 SNPs while allowing a maximum gap of 0.25 cM between SNPs and one heterozygote genotype per ROH using the command “–homozyg –homozyg‐window‐snp 25 –homozyg‐snp 25 –homozyg‐kb 390 –homozyg‐gap 250 –homozyg‐density 100 –homozyg‐window‐missing 2 –homozyg‐het 2 –homozyg‐window‐het 2.” We semi‐arbitrarily chose 0.39 cM as the minimum ROH length, which is the expected length of an ROH when the underlying haplotypes have a MRCA 128 generations ago as calculated with 100/(2g) cM (Thompson, 2013). Based on our SNP density, a stretch of genome with length 0.39 cM will contain on average ∼50 SNPs, which, together with a slow LD decay in Soay sheep (Stoffel et al., 2021b) should be sufficient to reliably call ROH of that length and above. To capture biologically interesting time horizons, we qualitatively assessed the distribution of ROH lengths in the population (Fig. S1), and subsequently clustered ROH into three length classes: long ROH (>12.5 cM) with an expected MRCA up to four generations ago and therefore likely to have originated from close inbreeding, medium ROH (1.56–12.5 cM) originating between 4 and 32 generations ago and reflecting the recent demographic history of the population and short ROH (0.39–1.56 cM) with an expected MRCA between 32 and 128 generations ago, reflecting deeper processes in the population history. For each length class, we calculated individual inbreeding coefficients *F*_ROH_ by summing up their total ROH length in each individual and dividing this value by the total sex‐averaged autosomal map length of 3146 cM. This can be thought of as a genetic map equivalent to the usual physical map based inbreeding coefficient *F*_ROH_ (Kardos et al., 2018). This resulted in three inbreeding coefficients per individual, *F*_ROHlong_, *F*_ROHmedium_, and *F*_ROHshort_, each ranging between 0 and 1.

### GENETIC SIMULATIONS

We used simulations to generate baseline expectations for how ROH length classes are expected to differ in their mutation load, and how this is influenced by different selection and dominance coefficients underlying new deleterious mutations. Specifically, we used forward genetic Wright‐Fisher simulations in SLiM 3 (Haller and Messer, 2019) to simulate deleterious mutations and overlaid neutral mutations using msprime (Kelleher, Etheridge, and McVean, 2016) and pyslim (Haller et al., 2019).

The Soay sheep were transferred to the St. Kilda archipelago around 4000 years or roughly 1000 generations ago (Clutton‐Brock and Pemberton, 2004), and their recent *N*_e_ has been estimated at 194 (Kijas et al., 2012). We simulated a population with a demographic history close to that estimated for Soay sheep, with a larger ancestral population size *N*_anc_ = 1000 for a period of 10,000 generations, followed by an instantaneous change to 200 individuals (after arrival on St. Kilda) for 1000 generations. Starting 30 generations in the past (at generation 10,970), we simulated an instantaneous bottleneck down to 10 individuals followed by an exponential recovery to 200 individuals within 20 generations to reflect the bottleneck due to the recent transfer of 107 sheep (22 of which were castrates) from the island of Soay to the island of Hirta in 1932, and their rapid population increase to between 600 and 2200 individuals nowadays. This broadly assumes a ratio of effective to census population size of 1:10, which is in line with Soay sheep *N*_e_ estimated from genomic data (Kijas et al., 2012).

We modeled 100 Mb diploid genomes with a uniformly distributed recombination rate of 1 × 10^−8^ per base pair per generation, so that the physical distance between two SNPs in Mb was on average equal to their genetic map distance in cM. In each generation, mutations were simulated at a rate of 1 × 10^−8^ per site, with 30% neutral mutations and 70% deleterious mutations (Kim, Huber, and Lohmueller, 2017). We explored the impact of different parameters underlying the distribution of fitness effects (DFE) for new deleterious mutations by simulating a range of selection and dominance coefficients. Specifically, selection coefficients *s* were drawn from gamma distributions with varying mean *s* ∈ {−0.01, −0.03, −0.05} and a shape parameter of 0.2, based on values estimated in humans (Eyre‐Walker, Woolfit, and Phelps, 2006). We also varied the dominance coefficients *h* for deleterious alleles from fully to partially recessive with *h* ∈ {0, 0.05, 0.2}. SLiM defines a mutation's fitness effect when homozygous as 1 + *s* and when heterozygous as *h*(1 + *h*s). Overall, we ran nine simulations for all combinations of *s* and *h*, with 50 replicates each.

At the end of each SLiM simulation, we generated a list of segregating deleterious mutations for the 200 individuals and saved the full tree sequence of the simulation (Haller et al., 2019). Neutral mutations were then added using the coalescent simulator msprime (Kelleher et al., 2016) and pyslim (Haller et al., 2019) and the results for each simulation were saved as vcf files. Before adding neutral mutations, we used recapitation, a technique which runs a coalescent simulation back in time to ensure the coalescence of all samples (Haller et al., 2019). We then called ROH in PLINK with the same parameters as in the empirical data analysis above, and clustered ROH into the same three ROH length classes.

Lastly, we combined ROH information with the deleterious mutation data and calculated the following three statistics, all of them as averages across all individuals within a given simulation: (1) The mutation load per cM ROH length. We defined the mutation load per unit length (in cM) for each ROH length class within an individual with $\frac{\sum_{1}^{n}s}{\sum_{1}^{m}\mathrm{ROH}\;\left(\mathrm{cM}\right)}$, where the numerator sums up the selection coefficients *s* for all *n* deleterious mutations found in the respective ROH class, and the denominator sums up the genetic map lengths of all *m* ROH segments in the relevant class within an individual. This measure of mutation load therefore quantifies the average expected fitness decline per cM of ROH; (2) the average number of deleterious mutations within each ROH class, per cM length; (3) the average allele frequency of deleterious mutations within each ROH class in the population. Lastly, in addition to mutation load, we also calculated a measure of inbreeding load across ROH classes, for which the general patterns were same as for mutation load (Supporting Information Analysis 1). We therefore report only the mutation load results in the main text.

### STATISTICAL ANALYSES

To estimate the effects of the three inbreeding coefficients *F*_ROHlong,_ *F*_ROHmedium_, and *F*_ROHshort_ on survival, we fitted a binomial Bayesian generalized linear mixed model with logit link, using brms (Bürkner, 2017), a high‐level R interface to Stan (Carpenter et al., 2017). The response variable was first‐year survival, with a value of 1 if a sheep survived to April 30 in the year after it was born and a value of 0 if it died. We used the following model structure:

Pr survi=1=logit−1(β0+FROHlongiβ1+FROHmediumiβ2+FROHshortiβ3+sexiβ4+twiniβ5+αkbirthyear+αlmotheridαkbirthyear∼N0,σbirthyear2,fork=1,⋯,39αlmotherid∼N0,σmotherid2,forl=1,⋯,1118.

The probability of survival for observation *i* ($\mathrm{Pr}(\;surv_{i}=\;1)$) was modeled with an intercept $\beta_{0}$, the three population level (fixed) effects for individual inbreeding coefficients *F*_ROH_ calculated from long ROH (>12.5 cM), medium ROH (between 1.56 and 12.5 cM), and short ROH (<1.56 cM), and two further population level effects to take into account the sex of the individual (female = 0, male = 1) and whether it was a twin (no = 0, yes = 1). The model also included two group‐level (random) intercept effects for birth year and maternal identity to model environmental variation across years and maternal effects, respectively. The three *F*_ROH_ variables were multiplied by 100, such that the model estimates the change in the odds of survival for a 1% increase in genomic ROH of the respective class. We used a normal prior with mean = 0 and SD = 5 for population‐level effects and the default half Student‐*t* prior for the standard deviation of group‐level parameters. We ran four MCMC chains with the NUTS sampler with 10,000 iterations each, a warmup of 5000 iterations and no thinning. All chains were visually checked for convergence and the Gelman‐Rubin criterion was <1.1 for all predictors, indicating good convergence (Gelman and Rubin, 1992). We present model estimates as odds ratios (ORs), which represent the predicted multiplicative change in the odds of survival for a unit increase in a given predictor, and 95% credible intervals based on the 2.5th and 97.5th percentile in the posterior distribution.

## Results

### ROH IN SOAY SHEEP

**Figure 1:**
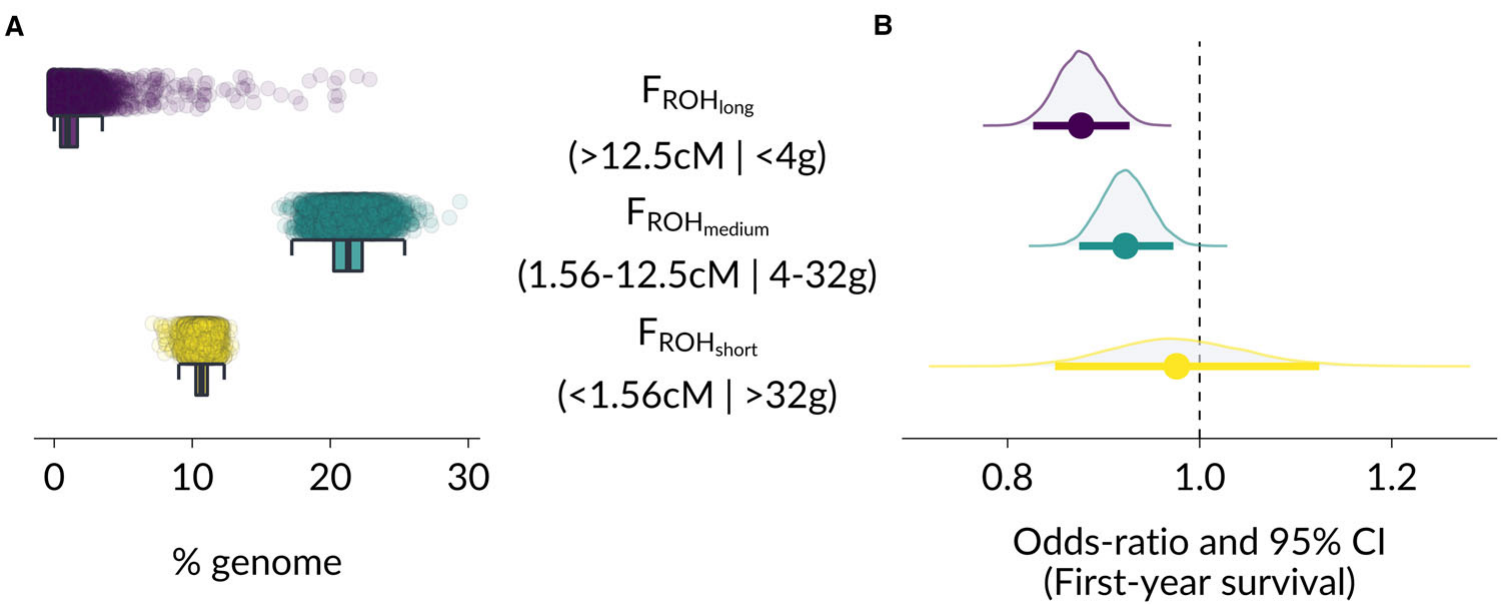
Distribution and fitness effects of inbreeding coefficients *F*_ROH_ based on different ROH lengths in Soay sheep. Panel (A) shows the distribution of *F*_ROHlong_, *F*_ROHmedium_, and *F*_ROHshort_ in the population, which were multiplied by 100 to represent the percentage of the genome in the respective ROH length class. Panel (B) shows the model estimates for the effects of the three inbreeding coefficients on first‐year survival. The effects are presented as odds ratios, which show the estimated multiplicative change in the odds of survival for a 1% genomic increase in the respective ROH class. The three classes were clustered by their genetic map length in cM, which is associated with the expected time to a MRCA in generations (g).

**Figure 2:**
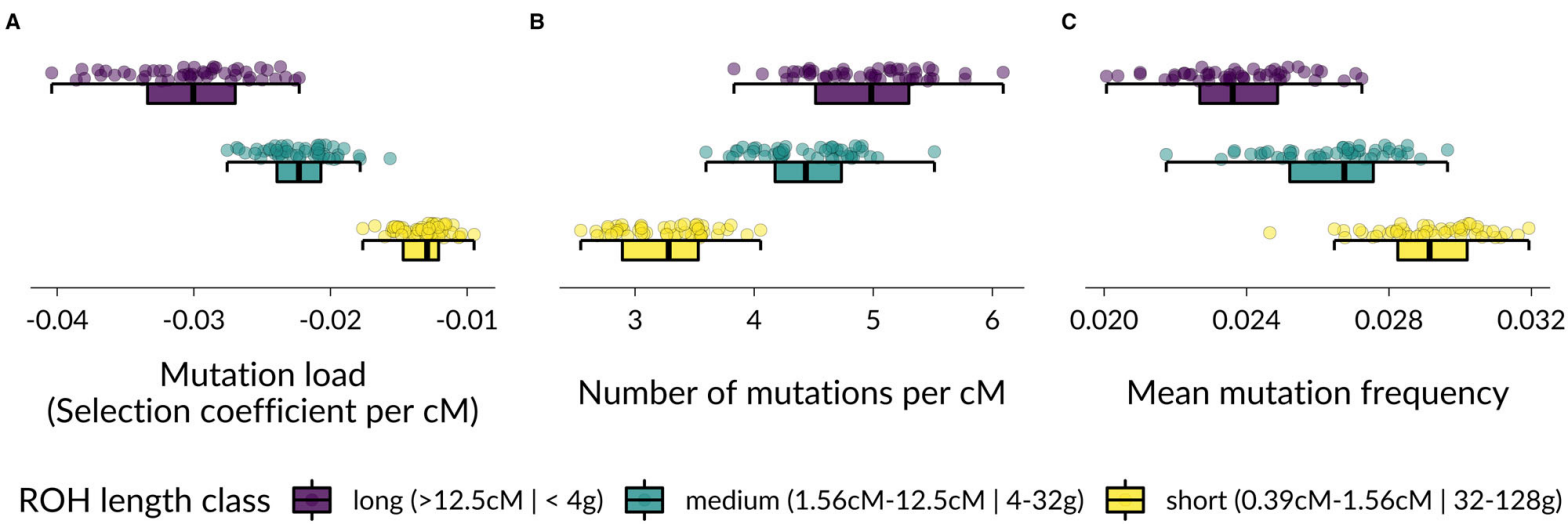
Patterns of deleterious mutations in long, medium, and short ROH. Each point represents the mean of 200 individuals of a simulation run. Panel (A) shows the mean selection coefficient of ROH per cM length, with lower values translating into a larger reduction in individual fitness and therefore representing a higher mutation load. Panel (B) shows the mean number of deleterious mutations per cM ROH length. Panel (C) shows the average population frequencies of deleterious mutations.

Overall, we quantified a total of 4,806,614 ROH across all 4879 Soay sheep, with a mean and maximum genetic map lengths of 1.68 and 80.55 cM, respectively. Individual sheep had on average 625 ROH (range 470–839) spanning 33.3% (range 27.8%–58.4%) of the autosomal genetic map. Initially, we visually assessed the distribution of ROH lengths over many classes (Fig. S1), and eventually clustered them into long, medium, and short ROH suitable for modeling (Fig. 1A). We calculated three individual inbreeding coefficients *F*_ROHlong_, *F*_ROHmedium_, and *F*_ROHshort_ based on these three ROH classes, which varied markedly in their means and distribution in the population (Figs. 1A and S1). Long ROH made up only 1.3% of the average Soay sheep genome (mean *F*_ROHlong_ = 0.013), though the distribution is right skewed and shows that long ROH added up to over 20% of the genome in the most inbred individuals. Medium ROH were the most common class in Soay sheep and made up 21.3% of the average autosomal genome, whereas short ROH made up 10.7% on average. A comparison between *F*_ROH_ based on the genetic map and *F*_ROH_ based on the physical map across individuals showed that the overall Pearson correlation was high (*R* = 0.96) and decreased for shorter ROH (*R* (*F*_ROHlong_) = 0.92, *R* (*F*_ROHmedium_) = 0.77, *R* (*F*_ROHshort_) = 0.64; Fig. 2).

### INBREEDING DEPRESSION IN SURVIVAL BY ROH LENGTH

Inbreeding depression was stronger when *F*_ROH_ was based on longer ROH (Fig. 1B and Table S1). The posterior mean OR for *F*_ROHlong_ was 0.876 (95% CI [0.827‐0.927]), or an estimated 12.4 % reduction in the odds of survival for a 1% increase in the proportion of the genome found within long ROH. For *F*_ROHmedium_, the OR was 0.923 (95% CI [0.875‐0.973]), corresponding to only a 7.7% reduction in the odds of survival for the same increase in ROH, and *F*_ROHshort_ were not associated with differences in survival (OR 0.977, 95% CI [0.850‐1.125]). In addition, the posterior distributions of the differences in model estimates for *F*_ROHlong_, *F*_ROHmedium_, and *F*_ROHshort_ are also reflecting differences in the estimated effects of inbreeding depression among ROH length classes; Fig. S3). Lastly, we fitted an alternative model, replacing the three *F*_ROH_ predictors with the overall inbreeding coefficient *F*_ROH_ and a second predictor quantifying the mean ROH length per individual. For a given overall inbreeding coefficient *F*_ROH_, a 1 cM increase in mean ROH length led to an estimated reduction in the odds of survival by 71% (OR 0.287, 95% CI [0.094‐0.874]), again reflecting stronger inbreeding depression in longer ROH (Table S2).

### GENETIC SIMULATIONS

To generate baseline expectations and insights into the reasons for differences in inbreeding depression between ROH length classes we used forward genetic simulations. The overall patterns were qualitatively similar for a range of selection and dominance coefficients for new deleterious mutations (Figs. S4−S6). Therefore, we focus here on the results of simulations based on deleterious mutations following a gamma DFE, with mean *s* = −0.03 and shape parameter *ß* = 0.2, where all mutations were partially recessive with a dominance coefficient *h* = 0.05 (Fig. 2). Long ROH had the highest overall mutation load per cM length, which was on average 26% lower in medium ROH and 56% lower in short ROH (Fig. 2A). The average frequency of deleterious mutations was lower in long compared to short ROH, showing that longer ROH are enriched for rarer deleterious mutations (Fig. 2C). The simulations also reveal a more nuanced pattern: Although the overall mutation load per cM was 26% lower in medium compared to long ROH, the average number of deleterious mutations was only 10% lower (Fig. 2B). This pattern emerges because rare, strongly deleterious mutations are quickly removed by purifying selection, leading to a substantially lower mutation load in haplotypes originating 4–32 generations ago compared to haplotypes originating less than four generations ago. Short ROH (<1.56 cM), with a MRCA more than 32 generations ago had the lowest mutation load and contained substantially fewer deleterious mutation with higher average frequencies (Fig. 2).

## Discussion

Long ROH originating from young haplotypes caused stronger inbreeding depression and had a higher mutation load than shorter ROH, which is expected when purifying selection acting over more generations has had more time to purge deleterious variation in older haplotypes. A substantial part of the mutation load is purged within tens of generations, causing a difference in inbreeding depression estimated from medium and long ROH, respectively. Our simulations suggest that this is likely due to selection against strongly deleterious mutations present at low frequencies. While this is theoretically expected in small populations (Kimura, Maruyama, and Crow, 1963; Wang et al., 1999; Hedrick and Garcia‐Dorado, 2016), empirical evidence based on actual fitness data is rare. However, deleterious mutations can be predicted from genome‐sequence data, which has revealed patterns of population‐wide purging due to bottlenecks and small population sizes in Mountain Gorillas, Isle Royale Wolves and Alpine Ibex (Xue et al., 2015; Robinson et al., 2019; Grossen et al., 2020). In Soay sheep, the difference in inbreeding depression between long and short ROH despite thousands of years of isolation as a small population suggests that intermediate and strongly deleterious mutations are unlikely to have been completely purged from the population. Instead, these differences probably reflect a haplotype‐level snapshot of the ongoing balance between newly arising strongly deleterious mutations expressed in long ROH, and selection against these mutations leaving shorter ROH with a lower mutation load.

Our findings have methodological implications for quantifying inbreeding depression and understanding its genetic architecture. Studies of wild animal population commonly use reduced representation methods such as SNP arrays or RAD sequencing for genotyping individuals. SNP densities might therefore not be high enough to reliably detect short ROH. In Soay sheep, most variation in inbreeding depression was captured by medium and long ROH, which can usually be reliably detected with intermediate SNP densities. Accordingly, we found that the effect of overall *F*_ROH_ on survival was similar when ROH were called from the original 38k SNPs compared to 417k imputed SNPs (Table S3). In studies of inbreeding depression in wild organisms with low *N*_e_ and high linkage disequilibrium, resources might therefore be better allocated into increasing the number of individuals rather than increasing SNP densities from tens of thousands of SNPs to whole‐genome sequencing. When studying the genetic basis of inbreeding depression, ROH can also be used to map the underlying loci in GWAS (Pryce et al., 2014; Kardos et al., 2016; Stoffel et al., 2021b). Our results suggest that the minimum ROH length is important when mapping ROH‐fitness relationships. When comparing the fitness of individuals with and without ROH at a given genomic location, the statistical power will be highest when only longer ROH are included, as these are more likely to harbor strongly deleterious recessive alleles. Analyses of the effects of different ROH length classes on fitness prior to GWAS analyses could therefore help to determine an optimal minimum ROH length.

Finally, our analyses provide some fundamental insights into the relationship between deleterious variation, inbreeding depression and purging in a small, wild population. At the haplotype level, we showed that purifying selection constantly removes deleterious variation, causing a difference in the mutation load of IBD haplotypes with different coalescent times. Strongly deleterious mutations are purged relatively quickly, probably because they frequently occur as homozygotes in small populations, which facilitate purging of mutations with large fitness effects despite a relatively low efficiency of selection due to drift (Hedrick and Garcia‐Dorado, 2016; Kyriazis, Wayne, and Lohmueller, 2021). Yet, inbreeding depression is strong in Soay sheep, and highly inbred individuals are very unlikely to survive their first winter (Fig. 1B, Table S3a, 2021b). Consequently, inbreeding depression in Soay sheep is probably largely a consequence of the combined effects of many weakly recessive deleterious alleles, which is consistent with a GWAS on the genetic basis of inbreeding depression in Soay sheep (Stoffel et al., 2021b). In small populations, theory predicts weakly deleterious mutations will drift more often to higher frequencies and fixation, thereby increasing the mutation load and decreasing mean fitness (Kimura et al., 1963). However, this also decreases the variance in deleterious mutations between individuals and therefore the expected strength of inbreeding depression (Hedrick and Garcia‐Dorado, 2016), which is why larger populations are predicted to experience even stronger inbreeding depression than for example Soay sheep. Combining subgenome level information such as ROH with fitness data is key to assess these theoretical predictions and to gain a deeper understanding of the genetic basis and strength of inbreeding depression in wild populations.

## AUTHOR CONTRIBUTIONS

JMP and MAS designed the study. JGP is the main Soay sheep project fieldworker and collected samples and life history data. JMP has run the Soay sheep long‐term study and organized the SNP genotyping. SEJ built the core genomic database, including genotyping, quality control and linkage mapping. MAS conducted data analyses and drafted the manuscript. MAS, JMP and SEJ jointly contributed to concepts, ideas and revisions of the manuscript.

## DATA ARCHIVING

All data underlying the analyses are publicly available on Zenodo (Stoffel et al., 2021a). The analysis scripts are available on GitHub (https://github.com/mastoffel/sheep_roh).

Associate Editor: A. Charmantier

## Supporting information


**Supplementary Figure 1**: Distribution of different ROH lengths classes in Soay sheep.
**Supplementary Figure 2**: Correlation between genetic and physical map *F*_ROH_.
**Supplementary Figure 3**: Posterior distribution for the differences in effect size estimates for inbreeding depression due to long, medium and short ROH.
**Supplementary Figure 4**: Mutation load (selection coefficient per cM) within ROH length classes for different DFE parameters.
**Supplementary Figure 5**: Abundance of deleterious mutations within ROH length classes for different DFE parameters.
**Supplementary Figure 6**: Deleterious mutation frequencies within ROH length classes for different DFE parameters.
**Supplementary Table 1**: Model estimates for a Bayesian animal model of juvenile survival with binomial error structure and logit link.
**Supplementary Table 2**: Model estimates for an alternative Bayesian animal model of juvenile survival with binomial error structure and logit link.
**Supplementary Table 3**: Effects of genome‐wide inbreeding *F*_ROH_ on juvenile survival with ROH called from two different SNP densities.
**Supplementary Figure 7**: Inbreeding load within ROH length classes for different DFE parameters.
