# Supplementary material for: Mutation load decreases with haplotype age in wild Soay sheep
Source: Evol Lett. 2021 May 17;5(3):187–95. doi: 10.1002/evl3.229 (PMC8190445; doi:10.1002/evl3.229)
Supplement: Supplementary file 1 — Supplementary Figure 1: Distribution of different ROH lengths classes in Soay sheep. Supplementary Figure 2: Correlation between genetic and physical map F ROH. Supplementary Figure 3: Posterior distribution for the differences in effect size estimates for inbreeding depression due to long, medium and short ROH. Supplementary Figure 4: Mutation load (selection coefficient per cM) within ROH length classes for different DFE parameters. Supplementary Figure 5: Abundance of deleterious mutations within ROH length classes for different DFE parameters. Supplementary Figure 6: Deleterious mutation frequencies within ROH length classes for different DFE parameters. Supplementary Table 1: Model estimates for a Bayesian animal model of juvenile survival with binomial error structure and logit link. Supplementary Table 2: Model estimates for an alternative Bayesian animal model of juvenile survival with binomial error structure and logit link. Supplementary Table 3: Effects of genome‐wide inbreeding F ROH on juvenile survival with ROH called from two different SNP densities. Supplementary Figure 7: Inbreeding load within ROH length classes for different DFE parameters. [file EVL3-5-187-s001.pdf]

Supplementary Material

Mutation load decreases with haplotype age in wild Soay sheep

Authors names and addresses:

Stoffel, M.A.[1*], Johnston, S.E.[1], Pilkington, J.G.[1], Pemberton, J.M[1]

[1]Institute of Evolutionary Biology, School of Biological Sciences, University of Edinburgh, Edinburgh, EH9 3FL, United Kingdom

* Corresponding author:
Martin A. Stoffel
Postal address: Institute of Evolutionary Biology, University of Edinburgh, Edinburgh, EH9 3FL, UK

E-mail: martin.stoffel@ed.ac.uk

Supplementary Figures

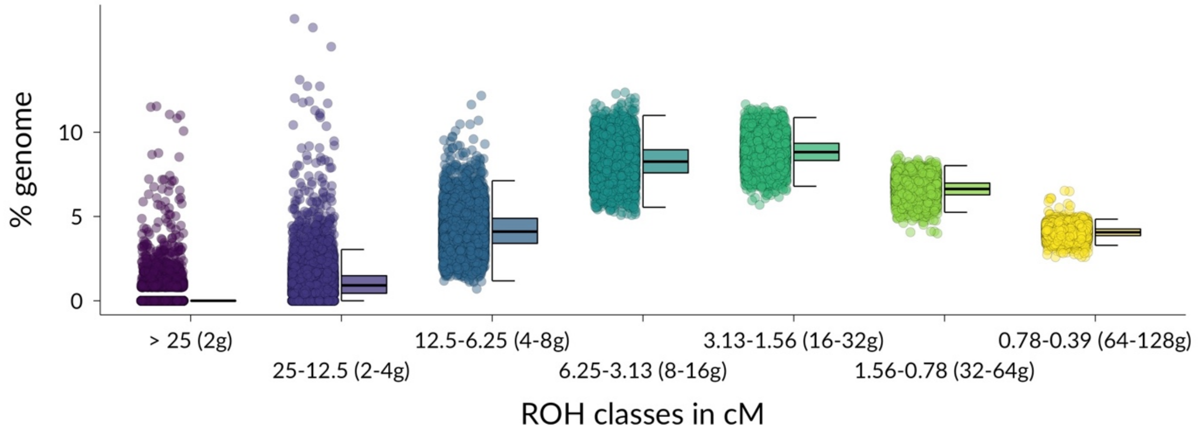

Supplementary Figure 1: Distribution of different ROH lengths classes in Soay sheep. ROH were measured in cM and clustered by their expected time to most recent common ancestor ranging from 2 to 128 generations ago. Each point represents the proportion of ROH of a specific length class in the genome of an individual.

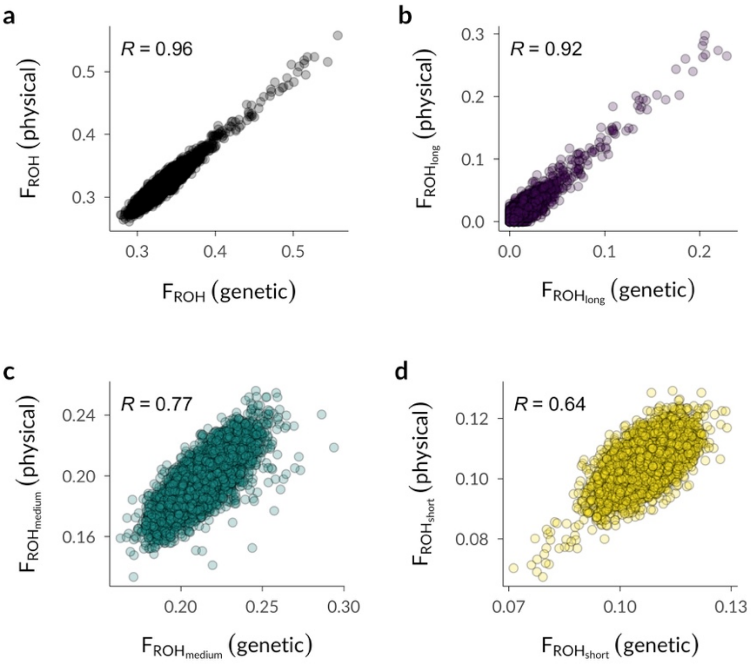

Supplementary Figure 2: Correlation between genetic and physical map F_{ROH}. Scatterplots of F_{ROH} based on genetically mapped ROH vs. F_{ROH} based on physically mapped ROH (using the same PLINK parameters) for **a** overall F_{ROH} **b** $F_{ROHlong}$ (ROH > 12.5 cM) **c** $F_{ROHmedium}$ (1.56 cM < ROH < 12.5) **d** $F_{ROHshort}$ (ROH < 1.56 cM). Each panel also shows the Pearson correlations (*R*).

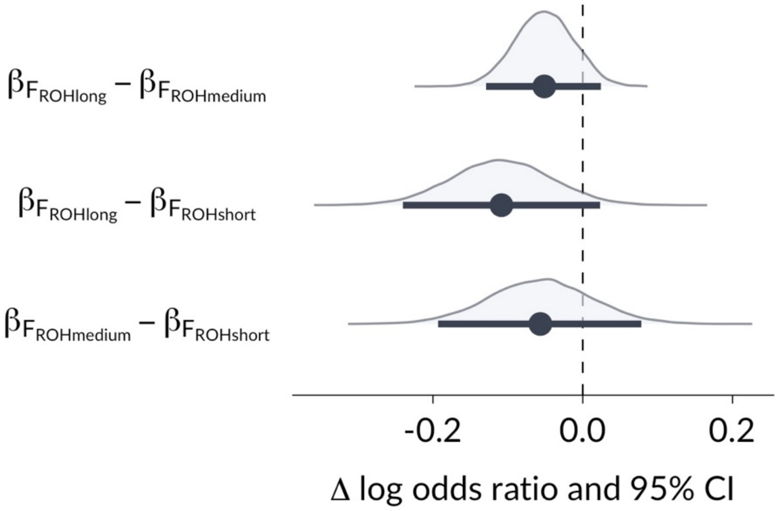

Supplementary Figure 3: Posterior distribution for the differences in effect size estimates for inbreeding depression due to long, medium and short ROH. In contrast to Figure 2a, the differences in model estimates are shown on the untransformed log-odds (logit) scale, which facilitates visualising the differences in model estimates for inbreeding depression based on the three different inbreeding coefficients. The posterior distributions are largely negative, indicating inbreeding depression estimates based longer ROH tended to have larger (negative) effect sizes.

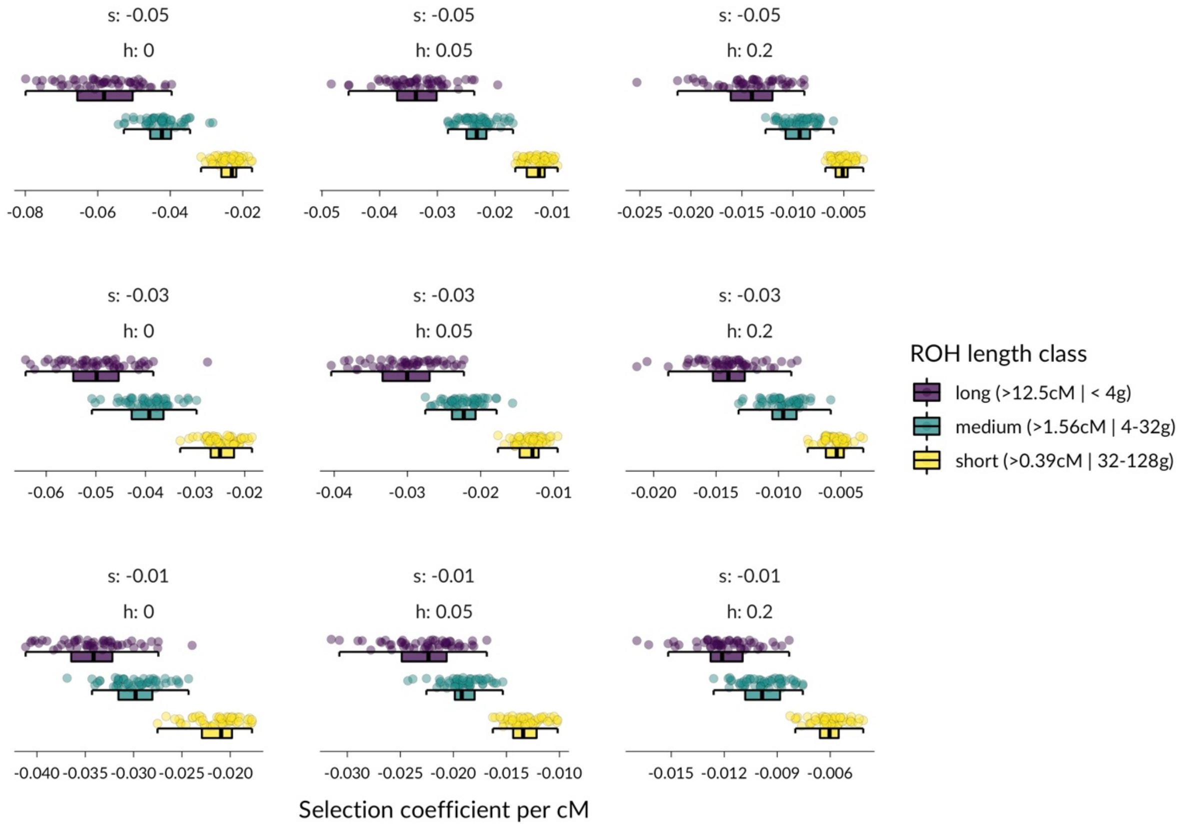

Supplementary Figure 4: Mutation load (selection coefficient per cM) within ROH length classes for different DFE parameters. Each datapoint represents the average selection coefficient per cM for three ROH length classes, calculated as the mean across 200 individuals sampled at the end of each simulation. The selection coefficients for new deleterious mutations were drawn from gamma distributions with mean s ∈ {-0.01,

-0.03, -0.05} and shape 0.2 and with three different dominance coefficients h ∈ {0, 0.05, 0.2}. The results of simulations with each combination of s and h are shown in the nine panels.

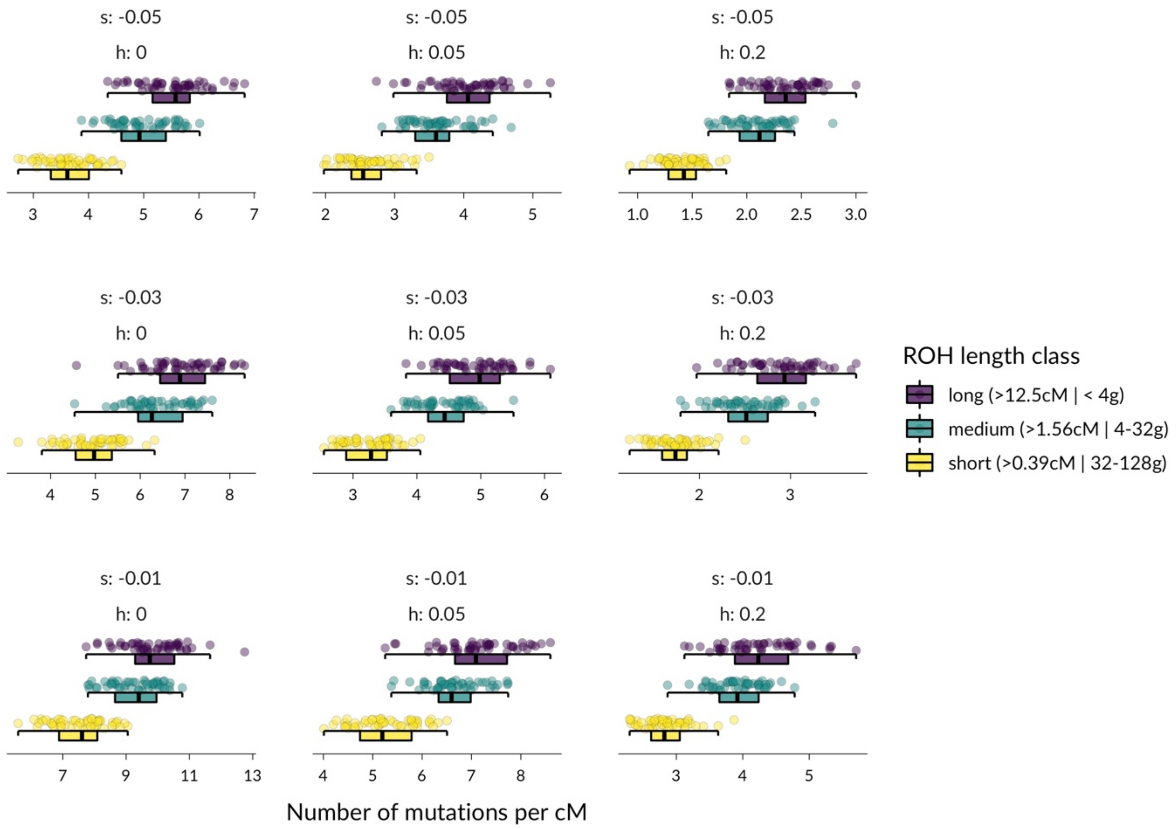

Supplementary Figure 5: Abundance of deleterious mutations within ROH length classes for different DFE parameters. Each datapoint represents the number of deleterious mutations per cM for three ROH length classes, calculated as the mean across 200 individuals sampled at the end of each simulation. The selection coefficients for new deleterious mutations were drawn from gamma distributions with mean s ∈ {-0.01, -0.03, -0.05} and shape 0.2 and with three different dominance coefficients h ∈ {0, 0.05, 0.2}. The results of simulations with each combination of mean s and h are shown in the nine panels.

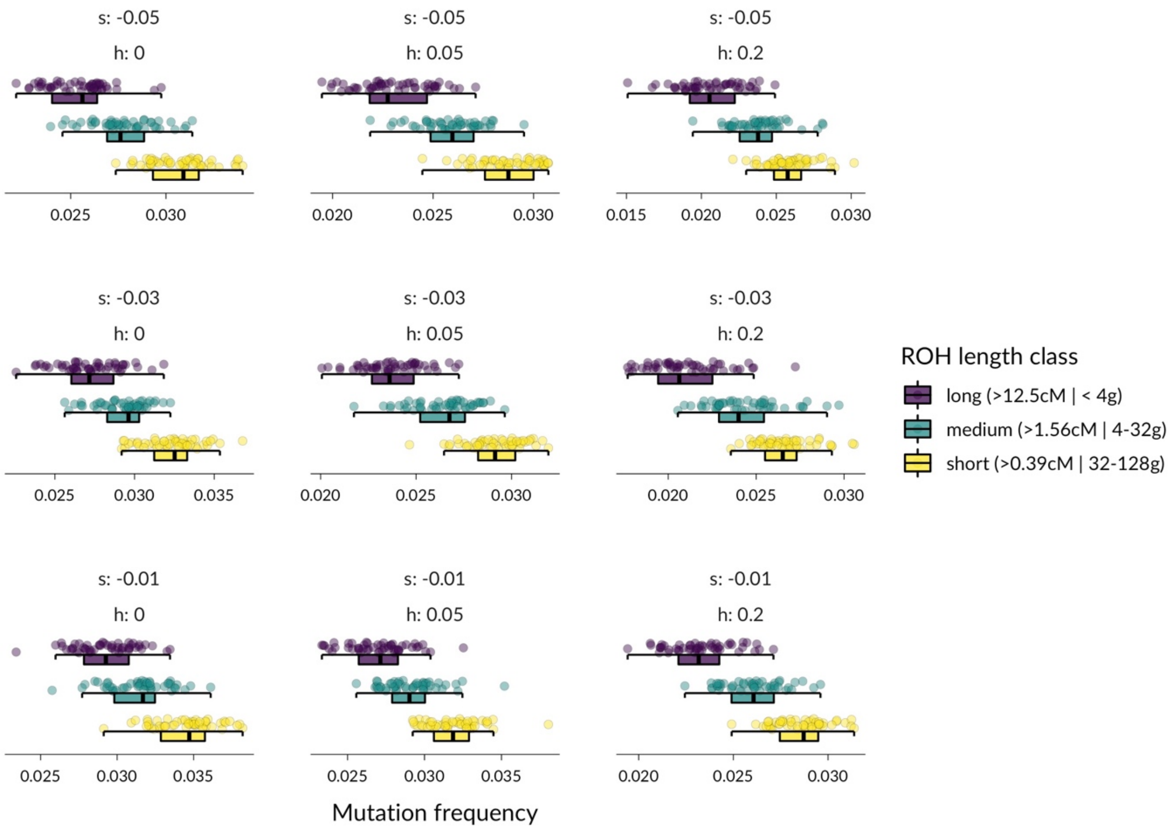

Supplementary Figure 6: Deleterious mutation frequencies within ROH length classes for different DFE parameters. Each datapoint represents the mean frequency of deleterious mutations contained in three ROH length classes, calculated as the mean across 200 individuals sampled at the end of each simulation. The selection coefficients for new deleterious mutations were drawn from gamma distributions with mean s ∈ {-0.01, -0.03, -0.05} and shape 0.2 and with three different dominance coefficients h ∈ {0, 0.05, 0.2}. The results of simulations with each combination of mean s and h are shown in the nine panels.

Supplementary Tables

Term	Post.Mean	CI (2.5%)	CI (97.5%)	Info
Intercept	2.423 (11.281)	0.214 (1.239)	4.658 (105.385)	
Population level/fixed effects				
$F_{ROHlong}$	-0.132 (0.876)	-0.19 (0.827)	-0.076 (0.927)	continuous
$F_{ROHmedium}$	-0.081 (0.923)	-0.134 (0.875)	-0.028 (0.973)	continuous
$F_{ROHshort}$	-0.024 (0.977)	-0.163 (0.85)	0.117 (1.125)	continuous
Sex	-0.67 (0.512)	-0.825 (0.438)	-0.519 (0.595)	categorical (0=male, 1=female)
Twin	-1.027 (0.358)	-1.242 (0.289)	-0.811 (0.445)	categorical (0=singleton, 1=twin)
Group level/random effects (standard deviation)				
Birth year	1.992 (7.33)	1.501 (4.486)	2.661 (14.306)	n = 1118
Mother ID	0.723 (2.061)	0.585 (1.795)	0.863 (2.369)	n = 39

Supplementary Table 1: Model estimates for a Bayesian animal model of juvenile survival with binomial error structure and logit link. Shown are the model estimates for the posterior mean and the lower and upper credible interval using the 2.5^{th} and 97.5^{th} quantile on both the logit scale and the odds-ratio scale in round brackets. The three F_{ROH} predictors have been multiplied by 100, so that the model directly estimates the log-odds change in survival for a 1% increase in genomic ROH. The last column shows the reference levels for the categorical predictors and the number of groups for group-level effects.

Term	Post.Mean	CI (2.5%)	CI (97.5%)	Info
Intercept	3.953 (52.108)	2.521 (12.437)	5.409 (223.51)	
Population level/fixed effects				
F_{ROH}	-0.047 (0.954)	-0.114 (0.892)	0.02 (1.02)	continuous
Mean ROH length (cM)	-1.248 (0.287)	-2.365 (0.094)	-0.135 (0.874)	continuous
Sex	-0.67 (0.512)	-0.823 (0.439)	-0.516 (0.597)	categorical (0=male, 1=female)
Twin	-1.026 (0.359)	-1.242 (0.289)	-0.813 (0.444)	categorical (0=singleton, 1=twin)
Group level/random effects (standard deviation)				
Birth year	1.988 (7.3)	1.496 (4.463)	2.655 (14.221)	n = 1118
Mother ID	0.717 (2.048)	0.578 (1.782)	0.855 (2.352)	n = 39

Supplementary Table 2: Model estimates for an alternative Bayesian animal model of juvenile survival with binomial error structure and logit link. Shown are the model estimates for the posterior mean and the lower and upper credible interval using the 2.5th and 97.5th quantile on both the logit scale and the odds-ratio scale in round brackets. The three F_{ROH} predictors have replaced by an overall inbreeding coefficient F_{ROH} including all ROH and a predictor quantifying the mean length of ROH within an individual. The negative fitness of ROH length shows that when keeping F_{ROH} constant, individuals with longer ROH have a lower survival probability. Note that F_{ROH} and Mean ROH length correlate highly (r = 0.85), which is why the estimate for F_{ROH} is relatively low. In addition, F_{ROH} has been multiplied by 100 again, so that the log-odds ratio and odds-ratio estimate a change in survival probability for a 1% increase in ROH.

a

Term	Post.Mean	CI (2.5%)	CI (97.5%)	Info
Intercept	3.905 (49.658)	2.486 (12.007)	5.294 (199.167)	
Population level/fixed effects				
F_{ROH}	-0.109 (0.897)	-0.146 (0.864)	-0.071 (0.931)	continuous
Sex	-0.67 (0.512)	-0.823 (0.439)	-0.515 (0.598)	categorical (0=male, 1=female)
Twin	-1.03 (0.357)	-1.247 (0.287)	-0.813 (0.444)	categorical (0=singleton, 1=twin)
Group level/random effects (standard deviation)				
Birth year	1.967 (7.152)	1.482 (4.401)	2.632 (13.905)	n = 1118
Mother ID	0.718 (2.051)	0.576 (1.779)	0.859 (2.361)	n = 39

b

Term	Post.Mean	CI (2.5%)	CI (97.5%)	Info
Intercept	1.524 (4.589)	0.653 (1.922)	2.41 (11.131)	
Population level/fixed effects				
F_{ROH}	-0.102 (0.903)	-0.151 (0.86)	-0.053 (0.948)	continuous
Sex	-0.667 (0.513)	-0.824 (0.439)	-0.512 (0.599)	categorical (0=male, 1=female)
Twin	-1.029 (0.357)	-1.246 (0.288)	-0.814 (0.443)	categorical (0=singleton, 1=twin)
Group level/random effects (standard deviation)				
Birth year	1.983 (7.264)	1.499 (4.476)	2.649 (14.142)	n = 1118
Mother ID	0.715 (2.044)	0.577 (1.781)	0.853 (2.347)	n = 39

Supplementary Table 3: Effects of genome-wide inbreeding F_{ROH} on juvenile survival with ROH called from two different SNP densities. We compared the survival effects of F_{ROH} when ROH where measured with **a** 417,373 imputed SNPs and **b** 38,130 original SNPs from the Illumina Ovine SNP50 BeadChip. To account for the lower SNP density and lower error rate in unimputed SNPs when calling ROH in PLINK using 38K SNPs, we set a higher minimum ROH length of 1.5 cM (compared to 0.39 cM in the imputed dataset), increased the possible gap between SNPs within an ROH to 0.5 cM (from 0.25 cM), and decreased allowed heterozygotes within ROH to 1 (from 2), while keeping all other parameters constant We used the following PLINK command to call ROH in from 38K SNPs: --homozyg --homozyg-window-snp 25 --homozyg-snp 25 --homozyg-kb 1500 --homozyg-gap 500 --homozyg-density 200 --homozyg-window-missing 2 --homozyg-het 1 --homozyg-window-het 1. Both tables show the model estimates for the posterior mean and the lower and upper credible interval using the 2.5th and 97.5th quantile on both the logit scale and the odds-ratio scale in round brackets. F_{ROH} was multiplied by 100, so that the model directly estimates the log-odds change in survival for a 1% increase in genomic ROH. The last column shows the reference levels for the categorical predictors and the number of groups for group-level effects.

Supplementary Analysis 1

As an alternative to quantifying the mutation load within ROH classes used in the main text, it is also possible to quantify a measure closer to the inbreeding load by comparing inbred to outbred fitness (Charlesworth & Charlesworth, 1987). To do this, we quantified the inbreeding load at each SNP location using $s_{ROH} - s_{pop}$, where s_{ROH} is the selection coefficient of the mutation within a focal ROH (i.e. an inbred individual). s_{pop} is the average selection coefficient at that site in the population, calculated as $p^2 + 2pq*(1+h*s) + (q^2 * 1+s)$, where q is the frequency of the deleterious allele, p is the frequency of the alternative allele, h is the dominance coefficient and s is the (negative) selection coefficient of the deleterious allele. The overall patterns are very similar to the patterns of mutation load within each ROH (Supplementary Figure 4). We therefore focused on mutation load in the main text.

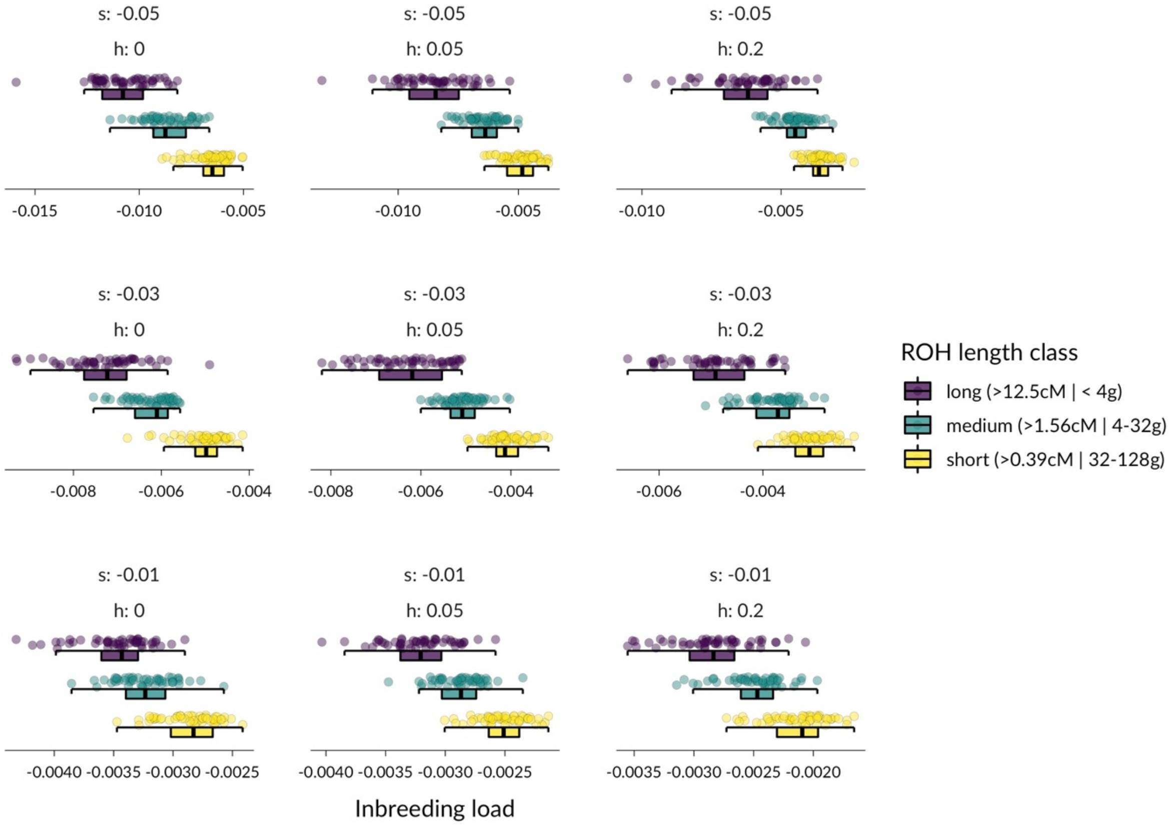

Supplementary Figure 7: Inbreeding load within ROH length classes for different DFE parameters. Each data point represents the average inbreeding load per site within each ROH length class within a simulation run. The selection coefficients for new deleterious mutations were drawn from gamma distributions with mean $s \in \{-0.01, -0.03, -0.05\}$ and a shape parameter of 0.2 and with three different dominance coefficients $h \in \{0, 0.05, 0.2\}$. The results of simulations with each combination of s and h are shown in the nine panels.

Literature

Charlesworth, D. & Charlesworth, B. (1987) Inbreeding Depression and Its Evolutionary Consequences. *Annual Review of Ecology and Systematics*, **18**, 237–268.
